# Efficacy of Post Exposure Administration of Doxycycline in a Murine Model of Inhalational Melioidosis

**DOI:** 10.1038/srep01146

**Published:** 2013-01-28

**Authors:** H. Carl Gelhaus, Michael S. Anderson, David A. Fisher, Michael T. Flavin, Ze-Qi Xu, Daniel C. Sanford

**Affiliations:** 1 Battelle, Battelle Memorial Institute, Columbus, OH, USA; 2 Advanced Life Sciences, Woodridge, IL, USA; 3 Current address: SynChem GMP, 1400 Chase Ave., Elk Grove Village, IL 60007

## Abstract

*Burkholderia pseudomallei* is the causative agent of melioidosis. Treatment of melioidosis is suboptimal and developing improved melioidosis therapies requires animal models. In this report, we exposed male BALB/c mice to various amounts of aerosolized *B. pseudomallei* 1026b to determine lethality. After establishing a median lethal dose (LD_50_) of 2,772 colony forming units (cfu)/animal, we tested the ability of doxycycline administered 6 hours after exposure to a uniformly lethal dose of ~20 LD_50_ to prevent death and eliminate bacteria from the lung and spleens. Tissue bacterial burdens were examined by PCR analysis. We found that 100% of mice treated with doxycycline survived and *B. pseudomallei* DNA was not amplified from the lungs or spleens of most surviving mice. We conclude the BALB/c mouse is a useful model of melioidosis. Furthermore, the data generated in this mouse model indicate that doxycycline is likely to be effective in post-exposure prophylaxis of melioidosis.

B*urkholderia pseudomallei* is a Gram-negative, motile, non-spore-forming, aerobic bacteria found in contaminated water, soil, and on market produce. It is the causative agent of melioidosis, an important infectious disease with highly endemic regions in southeast Asia and northern Australia and expanding known global distribution^1^,^2^. The primary reservoir of *B. pseudomallei* is the soil and water. Transmission from the environment to humans most commonly occurs via open wounds and skin abrasions as well as via inhalation during severe weather and can also occur via ingestion although this occurs more in animals than humans^2^,^3^,^4^. Melioidosis can manifest a broad range of symptoms, with both acute and chronic manifestations. Acute disease can be severe, including fever, weight loss, pneumonia, and death^5^. In addition to acute disease, asymptomatic chronic infections can exist for up to 62 years^6^. Although there is no documented history of the use of *B. pseudomallei* as a biological weapon, Ken Alibek claimed the Soviet Union weaponized *B. pseudomallei*^7^. Because of heightened concerns about the use of *B. pseudomallei* as a biological weapon, ongoing research is aimed at developing novel and safe anti-bacterial therapeutics.

*B. pseudomallei* is naturally resistant to a diversity of antibiotics, including penicillin, ampicillin, first and second generation cephalosporins, gentamicin, tobramycin, streptomycin, and polymixin^2^,^3^,^8^. Treatment involves an intensive intravenous infusion phase using either ceftazidime, meropenem, or imipenem for 10 to 14 days followed by a lengthy eradication phase of oral teimethoprim-sulfamethoxazole for up to 6 months^2^,^3^,^9^,^10^. Given the multidrug resistance of *B. pseudomallei* and lengthy treatment for melioidosis, there is a need to develop improved therapies. This requires well-characterized animal models of melioidosis with defined endpoints relevant to human disease for preclinical testing of new therapeutics and for screening of potential vaccine candidates. Additionally, given the low incidence of naturally acquired melioidosis in most parts of the world, the testing of new candidate vaccines and therapeutics may require demonstration of efficacy for licensure via the FDA Animal Rule (21 CFR 601.90–95). Therefore, the overall goal of our research is aimed at developing animal models to identify and evaluate treatments for melioidosis.

Numerous mouse models of *B. pseudomallei* infections have been developed, although most of these models used intranasal, intraperitoneal, or intravenous routes of infection, with LD_50_ ranging from < 5 to 1.5 × 10^4^ cfu/animal^11^,^12^,^13^,^14^,^15^,^16^,^17^. Differences in LD_50 _values are attributable to a number of factors include mouse strain, *B. pseudomallei* strain, and route of infection. Given the likelihood a biological weapon will utilize aerosol dissemination^18^ and the route of *B. pseudomallei* infection impacts disease manifestation^16^, an aerosol model has benefits not found in parenteral challenges. There have been recent efforts to develop mouse models of aerosolized *B. pseudomallei* infections^19^,^20^,^21^,^22^. We expand the nose-only exposure of male BALB/c mice to aerosolized *B. pseudomallei*, including the efficacy of post-exposure antibiotic administration. In particular, we examined doxycycline as it was used in older treatment protocols^23^, we have our experience with administering doxycycline treatments in mice^24^, and the vast majority of *B. pseudomallei* strains are susceptible to this antibiotic^25^,^26^,^27^,^28^. We report on the mouse model of inhalational melioidosis and the efficacy of post-exposure doxycycline administration in preventing the development of melioidosis.

## Results

### Lethality of the aerosol exposure

**Figure 1:**
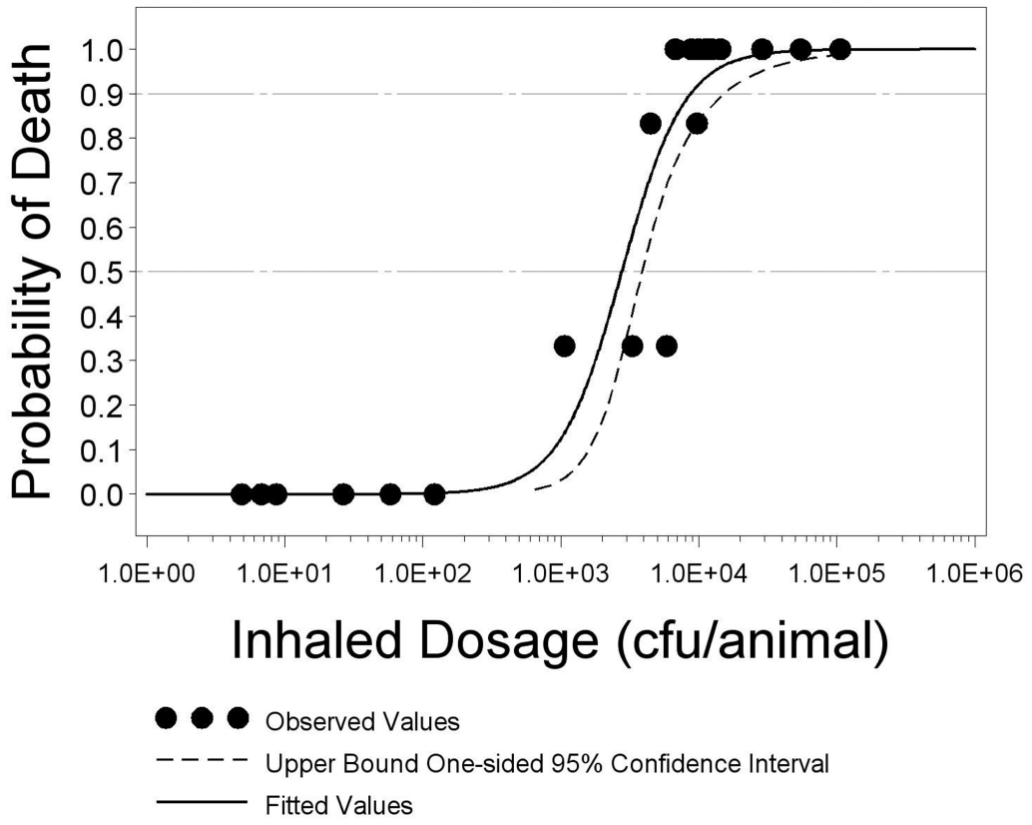
Estimated logistic regression curve and 95 percent confidence interval with points showing the proportion of animals that died. The probability of death of each group of male BALB/c mice is plotted against the inhaled dosage of *B. pseudomallei* 1026b in cfu. The percentage of animals alive 14 days after exposure to aerosolized *B. pseudomallei* 1026b in each dose group is depicted as ·. A logistic dose-response model was fit to the data and the solid black curve represents the results of this model, such that any *B. pseudomallei* 1026b dose has a corresponding probability of death. The dashed curve represents the upper 95% confidence bound of the logistic dose-response model. The horizontal dashed lines at 0.5 and 0.9 probability of death represent the median lethality and 90^th^ percentile of lethality, respectively. The points at which the solid black curve intersects the dashed horizontal lines represent the LD_50_ and LD_90_.

In order to have the greatest confidence in the LD_50_ estimate, we desired the maximum number of mice to be exposed to a dose near to the actual LD_50_. To best target the aerosol dose, mice were divided into three phases, with the targeted doses for phases II and III being determined by the cumulative lethality results from the previous phase(s). The targeted and actual aerosol doses are shown in Table 1. The average mass median aerodynamic diameter (MMAD) of the aerosol particles generated for phases I, II, and III was 2.34 μm, 2.03 μm, and 1.80 μm, respectively. The MMAD of the aerosol particles generated for this study are consistent with lower respiratory tract deposition. There were two animals (one in group I-E and one in group II-D) that were found dead immediately after removal from the aerosol exposure system and were excluded from all analyses. Analysis of these data found a statistically significant dose-response relationship (p = 0.0001) with higher dosages of the agent resulting in a higher probability of death. Figure 1 depicts the dose lethality curve. The LD_50_ was estimated at 2,772 cfu/animal with an upper 95 percent confidence bound of 3,888 cfu/animal. The LD_90_ was estimated to be 8,728 cfu/animal with an upper 95 percent confidence bound of 15,159 cfu/animal.

### Survival of mice with or without doxycycline

**Figure 2:**
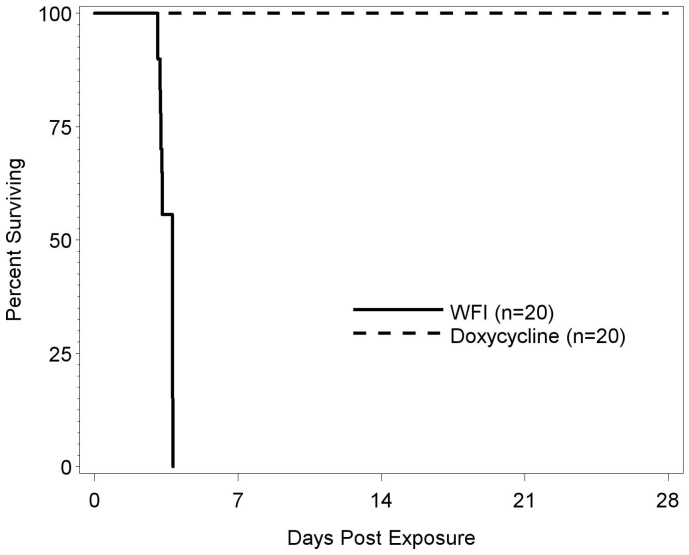
Survival of mice with or without doxycycline treatment following exposure to *B. pseudomallei* 1026b. Two groups of twenty male BALB/c mice were exposed to aerosolized *B. pseudomallei* 1026b via a nose-only exposure system. The animals were exposed to an inhaled dose range of 1.17 − 1.30 × 10^5^ cfu over a period of 10 minutes. Six hours after exposure, one group of mice was treated with doxycycline (dashed line) and the control group was treated with WFI (solid line). The 40 mg/kg doxycycline was administered twice daily for 14 days. The percentage of mice in each group surviving on each day following exposure to *B. pseudomallei* 1026b is shown.

Having established the LD_50_, we next wanted to examine the efficacy of doxycycline in preventing death following exposure to aerosolized *B. pseudomallei* 1026b. Mice inhaled an estimated 1.17 − 1.30 × 10^5^ cfu aerosolized B*. pseudomallei* 1026b. One group of mice was treated with doxycycline twice daily for 14 days beginning 6 hours after aerosol exposure. The control group received sterile water for injection on the same schedule. Figure 2 plots the Kaplan-Meier curves for the time-to-death data. All doxycycline treated mice survived 28 days after exposure to aerosolized *B. pseudomallei* 1026, while all water treated mice died 3–4 days after exposure. Fisher's exact comparing the survival rate between the two groups was performed and the differences in survival were significant (p < 0.0001).

### Impact of doxycycline on *B. pseudomallei* in tissues

**Figure 3:**
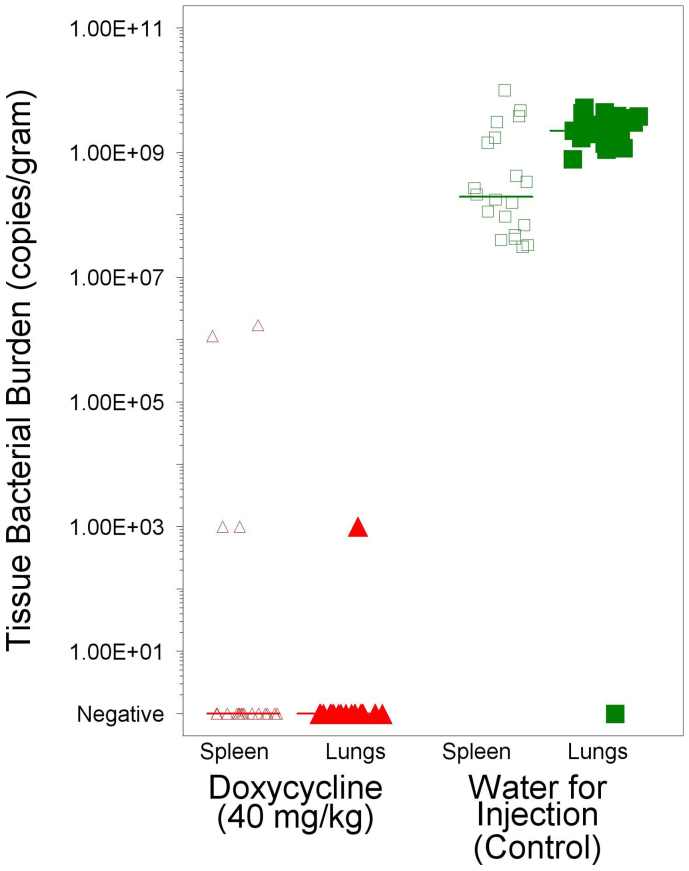
Tissue bacterial burden in the lungs and spleen. The copies of *B. pseudomallei* gene/g of tissue was determined for the spleen and lungs from each animal at the time of death or when euthanatized 28 days after exposure to *B. pseudomallei*. Copies/g were determined by real-time PCR and each symbol represents the results for an individual tissue. Triangle represent doxycycline treated mice and squares represent WFI treated controls. Open symbols represent spleen tissue results and filled symbols represent lung tissue results. The bars represent the mean copies/g in a given tissue for a given treatment group. Doxycycline treatment significantly reduced the copies/g in both the spleen and lungs (p < 0.0001).

As *B. pseudomallei* infections can manifest as either a lethal acute infection or a sub-lethal chronic infection, we wanted to assess the number of bacteria present in the lungs and spleens of animals at the time of death or at euthanasia 28 days after aerosol exposure. As shown in Figure 3, the specimens from mice found dead in the WFI treatment group had a median 1.95 × 10^8^ copies of *rpoB*/gram of spleen and 2.25 × 10^9^ copies of *rpoB*/gram of lung. In comparison, 16 of 20 spleen specimens and 19 of 20 lung specimens had no detectable copies of *rpoB*. These results indicate that doxycycline greatly reduced *B. pseudomallei* tissue burdens and likely eradicated infection in 80% of mice.

## Discussion

Similar to previous studies, we show that *B. pseudomallei* causes a lethal disease in BALB/c mice and that doxycycline can be used as a treatment. Doxycycline treatment resulted in no detectable bacteria in 19 of 20 lungs and 16 of 20 spleens. In previous reports, whole body exposure to aerosolized *B. pseudomallei* 1026b resulted in an LD_50_ 1 × 10^1^ cfu/mouse, well below 2.8 × 10^3^ we found for nose-only aerosol in male BALB/c mice. While this difference may be attributable to the differences in the *B. pseudomallei* aerosolized, differences in route of exposure (whole body vs. nose only), and sex differences also existed. It is important to note that we examined male BALB/c mice rather than females used in six previous reports^19^,^20^,^22^,^29^,^30^,^31^. It is worth noting that 70–75% of melioidosis cases are male, indicating a sex difference in melioidosis susceptibility^5^,^32^,^33^. However, it has been suggested that sex differences observed in clinical melioidosis may be due to increased exposure due to outdoor occupations of men^33^.

Doxycycline was effective at preventing death from exposure to aerosolized *B. pseudomallei*, similar to what was shown by Sivalingam et al., who demonstrated that 100% of mice survived when doxycycline was administered at the time of challenge and 80% of mice survived when doxycycline was administered 10 hours after challenge^34^. The sterility achieved in the lungs in 19 of 20 mice was similar to what was previously shown with no bacteria being detected by nine days post-challenge. In conclusion, the BALB/c mouse is a useful model of melioidosis and post-exposure prophylaxis by doxycycline is likely to be effective, based on the mouse model.

## Methods

### Aerosolization of *B. pseudomallei*

*B. pseudomallei* 1026b was obtained from BEI Resources (cat #NR-4074) and a working cell bank of single use glycerol stock vials are cryopreserved at −70°C at Battelle. The *B. pseudomallei* was propagated from frozen culture in Luria broth plus 4% glycerol. Mid to late-log phase cultures were used to generate aerosols. The aerosol exposure system used for the mouse aerosol challenge testing consisted of a system capable of exposing multiple animals (up to 30) with the addition of impinger samplers, an aerosol particle size analyzer, temperature and humidity monitoring, mass flow meters (MFM) and mass flow controllers (MFC) to monitor the aerosol flows. To generate *B. pseudomallei* 1026b containing aerosols, forced air entered Battelle's custom system through high efficiency particulate air (HEPA) filters and was divided into a continuous air stream (continuous air) and an air stream that either flowed into the Collison nebulizer (during aerosol generation) or by-passes it (between aerosol generations). Mass flow controllers (MFC) regulated the flow for each of the air streams. The *B. pseudomallei* aerosol, created by the nebulizer, was allowed to mix with continuous air before being delivered to the exposure chamber. A nose-only aerosol exposure system (CH Technologies Tower) was utilized to deliver the desired *B. pseudomallei* aerosol. From the exposure chamber the aerosol was sampled for concentration dose determination of *B. pseudomallei* using an impinger (model 7541, Ace Glass, Inc.). The liquid in the nebulizer and impinger was diluted and enumerated by the spread plate and/or filtering technique to quantify viable *B. pseudomallei* bacteria counts per mL. The *B. pseudomallei* concentration (based on enumerations) of the nebulizer and impinger samples were used to determine the actual exposure dose for each challenge group. Using Guyton's formula and the concentration of *B. pseudomallei* collected from air sampled from the exposure system, the inhaled dose was determined from the enumeration results.

### Animals

A total of 160 male BALB/c mice approximately 6–8 weeks of age were obtained from Charles River. Mice were obtained at different times for either the LD_50_ determination or for the efficacy of doxycycline determination. Mice were observed twice daily throughout the experiments for signs of morbidity and mortality. Animal use protocols were approved by Battelle's Institutional Animal Care and Use Committee (IACUC) and the Department of Defense Animal Care and Use Review Office (ACURO).

### LD_50_ determination

A total of 120 male BALB/c mice were used to estimate the LD_50_, in three phases. In phase I, eight groups of six mice were exposed to escalating targeted doses of aerosolized *B. pseudomallei* 1026b ranging from 5 to 5,000 cfu/mouse. Phase II consisted of six groups of six mice were exposed to escalating targeted doses of aerosolized *B. pseudomallei* 1026b ranging from 7,000 to 100,000 cfu/mouse. Phase III consisted of six groups of six mice were exposed to escalating targeted doses of aerosolized *B. pseudomallei* 1026b ranging from 2,000 to 8,000 cfu/mouse. LD_50_ estimates were made following each phase to plan targeted doses for the subsequent phase.

### Antibiotic efficacy testing

A total of 40 male BALB/c mice were used to test the efficacy of doxycycline in post-exposure prophylaxis. Mice were divided into doxycycline and sterile water for injection (WFI) groups of 20 mice each. Five mice from each group were exposed to aerosolized *B. pseudomallei* 1026b on four different runs to a target of 5.6 × 10^4^ cfu (~20 LD_50_). Following the aerosol challenge, antibiotic therapy began approximately 6 (±1 hr) hours post median challenge time. Mice in the doxycycline treatment group received doxycycline at a dose of 40 mg/kg twice a day (BID) for 14 days [every 12 (±1 hr) hours]. This dosing regimen was selected to ensure that the area under the concentration time curve for a 24 hour period (AUC_0–24_) was similar in mice compared to the human label dose. This approach is favored as the antimicrobial pattern of doxycyline predicts that AUC_0–24_ will determine efficacy^35^. Doxycycline dose label indicates 100 mg every 12 hours for the average person. However, published human data was only available for a 200 mg dose, with an AUC_0–12_ of 30 μg*h/mL (based on trapezoidal calculation using data from Newton 2005^36^). This data was used as the basis of a 100 mg twice daily regimen and calculated the AUC_0–24_ was approximately 30 μg*h/mL. In mice, the AUC_0–6_ has been reported to be 13.7 μg*h/mL with a dose of 25 mg/kg^37^. In order to achieve an AUC_0–24_ near 30 μg*h/mL, BID dosing of 40 mg/kg was used. Doxycycline (Vibramycin® calcium syrup oral suspension, Pfizer Labs, NY, NY) was obtained from a local pharmacy. Control mice received WFI for 14 days [every 24 (±1 hr) hours] or until death at a volume of 5 mL/kg QD**.** Dosing occurred via orogastric intubation. Mice were observed for clinical signs of disease and survival for 28 days following aerosol exposure.

### Tissue bacterial burden

The bacterial burden in the lungs and spleen was determined by quantitative PCR from all mice found dead or euthanized from the antibiotic efficacy testing. At the time a mouse was found dead or following euthanasia, a specimen of lung and spleen at gross necropsy was collected for bacterial burden analysis. The lung and spleen specimens were weighed, manually homogenized using a tissue grinder and total nucleic acid was isolated using the NucliSens easyMAG (bioMerieux, Durham, NC). Briefly, 100 μL of sample was added to sample vessels that were loaded into the instrument. The instrument then dispensed easyMAG Lysis Buffer (bioMerieux, Durham, NC) and incubated the samples for 10 minutes. Afterwards, easMAG Magnetic Silica (bioMerieux, Durham, NC) was added to the samples. The instrument then incubated the samples for 10 minutes, collected the silica into pellets, and washed them subsequently with easyMAG Extraction Buffer 1 (bioMerieux, Durham, NC), easyMAG Extraction Buffer 2 (bioMerieux, Durham, NC), and easyMAG Extraction Buffer 3 (bioMerieux, Durham, NC). The purified nucleic acid was then eluted from the silica with 100 μL of easyMAG Extraction Buffer 3(bioMerieux, Durham, NC) and assayed**.** Based on published sequence data, oligonucleotides were designed to amplify a small fragment within the coding region of the *rpoB* gene product on the *B. pseudomallei* chromosome. We selected the *rpoB* target because it contained a 1026b sequence that was not identified in other B. pseudomallei strains with previously published genome sequences. Thus, oligonucleotide sequences were submitted to Applied Biosystems for the generation of a TaqMan® Gene Expression Assay which generated a forward primer of 5'-CCGGCGGCAGTTCGT-3', a reverse primer of 5'-AGCTCGAAGCGATCAAGAACTC-3' and a probe of 6'FAM-CGAACTGGTGGCGCT-MGBNFQ-3′. Qualitative real-time PCR reactions consisted of 1× TaqMan® Gene Expression Master Mix [AmpliTaq Gold® DNA Polymerase, AmpErase® UNG, dNTPs with dUTP, Passive Reference, and optimized buffer components (Applied Biosystems Inc., Foster City, CA)], 1× Gene Expression Assay mixture [900 nM forward primer, 900 nM reverse primer, and 250 nM probe (dual-labeled with FAM^TM^ at the 5′ and a non-fluorescent quencher at the 3′ end)], dIH_2_0, and 5 μL isolated genomic DNA in a total volume of 50 μL. Qualitative real-time PCR was performed using an ABI PRISM® Sequence Detection System (Applied Biosystems Inc., Foster City, CA) with the following conditions: 2 min at 50°C, 10 min at 95°C, followed by 40 cycles of 95°C for 15 seconds and 60°C for 1 min. All reactions were performed in triplicate, and each run contained known negative (genomic DNA isolation procedure using dIH_2_0) and positive (genomic DNA isolated from a *B. pseudomallei* vegetative culture) controls. Following acquisition, data was analyzed using Version 2.3 of the Sequence Detection System software (Applied Biosystems Inc., Foster City, CA). A quantity was reported for any sample that demonstrated a quantity ≥ 2 copies/μL. For samples < 2 copies/μL, the sample was considered negative.

### Statistical methods

The cumulative data from all completed phases were fitted to a logistic regression model to obtain a preliminary estimate of the LD_50 _to define the next phase. Once all three phases were completed, all data were fitted to a logistic regression model whereby the probability of lethality was modeled against the base-10 logarithm of the inhaled dose. All models were fitted in SAS® (ver. 9.1) using PROC PROBIT with a logistic link function. Estimated parameters of the logistic model were used to compute the LD_50 _and LD_90_ while Fieller's method was used to compute an upper one-sided 95 percent confidence bound for each estimate.

For antibiotic efficacy testing, survival rates were calculated for each group. A two-sided Fisher's exact test was used to compare the survival rates between the groups. Then, a time-to-death model was fitted using the treatment and control group. The Kaplan-Meier curves were plotted and the log-rank test was computed to determine if the difference was statistically significant. To determine if there were significant differences in the distribution of tissue bacterial burden levels between the treated and control groups, a two-sample Kolmogorov-Smirnov test was performed on the tissue bacterial burden data for each specimen location.

## Author Contributions

H.C.G. and D.C.S. wrote the main manuscript text, M.S.A. designed and conducted statistical analysis and wrote the statistical methods section, D.A.F. designed and conducted the aerosol exposure, M.T.F., Z.X. and D.C.S. designed the studies. All authors reviewed the manuscript.

## Figures and Tables

**Table 1:** Lethality results

Phase	Dosage Group	No. of Animals	Target Challenge Dosage (cfu/animal)	Calculated Inhaled Dosage (cfu/animal)	No. of Animals Died
I	I-A	6	5	7	0
	I-B	6	10	9	0
	I-C	6	25	5	0
	I-D	6	50	58	0
	I-E	6	100	27	0
	I-F	5	500	122	0
	I-G	6	1,000	1,068	2
	I-H	6	5,000	5,854	2
II	II-A	6	7,000	8,910	6
	II-B	6	8,000	12,408	6
	II-C	6	10,000	9,966	6
	II-D	5	25,000	28,896	5
	II-E	6	50,000	54,824	6
	II-F	6	100,000	106,260	6
III	III-A	6	2,000	3,309	2
	III-B	6	3,000	4,488	5
	III-C	6	4,000	6,798	6
	III-D	6	5,000	9,725	5
	III-E	6	7,000	11,286	6
	III-F	6	8,000	14,424	6

## References

[b1] Currie B. J., Dance D. A. & Cheng A. C. The global distribution of Burkholderia pseudomallei and melioidosis: an update. Trans R Soc Trop Med Hyg **102** Suppl 1, S1–4 (2008) 19121666 10.1016/S0035-9203(08)70002-6

[b2] Wiersinga W. J., Currie B. J. & Peacock S. J. Melioidosis. N Engl J Med 367, 1035–44 (2012). 22970946 10.1056/NEJMra1204699

[b3] Cheng A. C. & Currie B. J. Melioidosis: epidemiology, pathophysiology, and management. Clin Microbiol Rev 18, 383–416 (2005). 15831829 10.1128/CMR.18.2.383-416.2005 PMC1082802

[b4] Currie B. J. & Jacups S. P. Intensity of rainfall and severity of melioidosis, Australia. Emerg Infect Dis 9, 1538–42 (2003). 14720392 10.3201/eid0912.020750 PMC3034332

[b5] Currie B. J., Ward L. & Cheng A. C. The epidemiology and clinical spectrum of melioidosis: 540 cases from the 20 year Darwin prospective study. PLoS Negl Trop Dis 4, e900 (2010). 21152057 10.1371/journal.pntd.0000900 PMC2994918

[b6] Ngauy V., Lemeshev Y., Sadkowski L. & Crawford G. Cutaneous melioidosis in a man who was taken as a prisoner of war by the Japanese during World War II. J Clin Microbiol 43, 970–2 (2005). 15695721 10.1128/JCM.43.2.970-972.2005 PMC548040

[b7] Alibek K. & Handelman S. Biohazard: The Chilling True Story of the Largest Covert Biological Weapons Program in the World--Told from Inside by the Man Who Ran It Random House, 1999.

[b8] Thibault F. M., Hernandez E., Vidal D. R., Girardet M. & Cavallo J. D. Antibiotic susceptibility of 65 isolates of Burkholderia pseudomallei and Burkholderia mallei to 35 antimicrobial agents. J Antimicrob Chemother 54, 1134–8 (2004). 15509614 10.1093/jac/dkh471

[b9] Currie B. J., Fisher D. A., Anstey N. M. & Jacups S. P. Melioidosis: acute and chronic disease, relapse and re-activation. Trans R Soc Trop Med Hyg 94, 301–4 (2000). 10975006 10.1016/s0035-9203(00)90333-x

[b10] White N. J. Melioidosis. Lancet 361, 1715–22 (2003). 12767750 10.1016/s0140-6736(03)13374-0

[b11] Veljanov D., Vesselinova A., Nikolova S., Najdenski H., Kussovski V. & Markova N. Experimental melioidosis in inbred mouse strains. Zentralbl Bakteriol 283, 351–9 (1996). 8861874 10.1016/s0934-8840(96)80071-5

[b12] Leakey A. K., Ulett G. C. & Hirst R. G. BALB/c and C57Bl/6 mice infected with virulent Burkholderia pseudomallei provide contrasting animal models for the acute and chronic forms of human melioidosis. Microb Pathog 24, 269–75 (1998). 9600859 10.1006/mpat.1997.0179

[b13] Hoppe I., Brenneke B., Rohde M., Kreft A., Haussler S., Reganzerowski A. & Steinmetz I. Characterization of a murine model of melioidosis: comparison of different strains of mice. Infect Immun 67, 2891–900 (1999). 10338496 10.1128/iai.67.6.2891-2900.1999 PMC96597

[b14] Santanirand P., Harley V. S., Dance D. A., Drasar B. S. & Bancroft G. J. Obligatory role of gamma interferon for host survival in a murine model of infection with Burkholderia pseudomallei. Infect Immun 67, 3593–600 (1999). 10377144 10.1128/iai.67.7.3593-3600.1999 PMC116549

[b15] Gauthier Y. P., Hagen R. M., Brochier G. S., Neubauer H., Splettstoesser W. D., Finke E. J. & Vidal D. R. Study on the pathophysiology of experimental Burkholderia pseudomallei infection in mice. FEMS Immunol Med Microbiol 30, 53–63 (2001). 11172992 10.1111/j.1574-695X.2001.tb01550.x

[b16] Liu B. Koo G. C., Yap E. H., Chua K. L. & Gan Y. H. Model of differential susceptibility to mucosal Burkholderia pseudomallei infection. Infect Immun 70, 504–11 (2002). 11796576 10.1128/IAI.70.2.504-511.2002 PMC127661

[b17] Van Zandt K. E. Tuanyok A. Keim P. S. Warren R. L. & Gelhaus H. C. An Objective Approach for Burkholderia pseudomallei Strain Selection as Challenge Material for Medical Countermeasures Efficacy Testing. Front Cell Infect Microbiol 2, 120 (2012). 23057010 10.3389/fcimb.2012.00120 PMC3458228

[b18] Roy C. J. & Pitt M. L. Infectious Disease Aerobiology: Aerosol Challenge Methods. in: J. R. Swearengen, (Ed.), Biodefense Research Methodology and Animal Models, CRC Press, Boca Raton, FL, 2006, pp. 61–76.

[b19] Lever M. S., Nelson M., Stagg A. J., Beedham R. J. & Simpson A. J. Experimental acute respiratory Burkholderia pseudomallei infection in BALB/c mice. Int J Exp Pathol 90, 16–25 (2009). 19200247 10.1111/j.1365-2613.2008.00619.x PMC2669612

[b20] Jeddeloh J. A., Fritz D. L., Waag D. M., Hartings J. M. & Andrews G. P. Biodefense-driven murine model of pneumonic melioidosis. Infect Immun 71, 584–7 (2003). 12496217 10.1128/IAI.71.1.584-587.2003 PMC143420

[b21] Nieves W., Asakrah S., Qazi O., Brown K. A., Kurtz J., Aucoin D. P., McLachlan J. B., Roy C. J. & Morici L. A. A naturally derived outer-membrane vesicle vaccine protects against lethal pulmonary Burkholderia pseudomallei infection. Vaccine 29, 8381–9 (2011). 21871517 10.1016/j.vaccine.2011.08.058 PMC3195868

[b22] Tan G. Y., Liu Y., Sivalingam S. P., Sim S. H., Wang D., Paucod J. C., Gauthier Y. & Ooi E. E. Burkholderia pseudomallei aerosol infection results in differential inflammatory responses in BALB/c and C57Bl/6 mice. J Med Microbiol 57, 508–15 (2008). 18349373 10.1099/jmm.0.47596-0

[b23] Chaowagul W., Chierakul W., Simpson A. J., Short J. M., Stepniewska K., Maharjan B., Rajchanuvong A., Busarawong D., Limmathurotsakul D., Cheng A. C., Wuthiekanun V., Newton P. N., White N. J., Day N. P. & Peacock S. J. Open-label randomized trial of oral trimethoprim-sulfamethoxazole, doxycycline, and chloramphenicol compared with trimethoprim-sulfamethoxazole and doxycycline for maintenance therapy of melioidosis. Antimicrob Agents Chemother 49, 4020–5 (2005). 16189075 10.1128/AAC.49.10.4020-4025.2005 PMC1251512

[b24] Gelhaus H. C. Barnewall R. Cross J. Lindsay A. S. & Lockman, H. Qualification of a Mouse Model of Tularemia by Confirming Doxycycline Efficacy, 50th Interscience Conference on Antimicrobial Agents and Chemotherapy, Boston, MA, 2010.

[b25] Kenny D. J., Russell P., Rogers D., Eley S. M. & Titball R. W. In vitro susceptibilities of Burkholderia mallei in comparison to those of other pathogenic Burkholderia spp. Antimicrob Agents Chemother 43, 2773–5 (1999). 10543761 10.1128/aac.43.11.2773 PMC89557

[b26] Jenney A. W., Lum G., Fisher D. A. & Currie B. J. Antibiotic susceptibility of Burkholderia pseudomallei from tropical northern Australia and implications for therapy of melioidosis. Int J Antimicrob Agents 17, 109–13 (2001). 11165114 10.1016/s0924-8579(00)00334-4

[b27] Fisher M. W., Hillegas A. B. & Nazeeri P. L. Susceptibility in vitro and in vivo of Pseudomonas pseudomallei to rifampin and tetracyclines. Appl Microbiol 22, 13–6 (1971). 5111303 10.1128/am.22.1.13-16.1971 PMC377368

[b28] Hall W. H. & Manion R. E. Antibiotic susceptibility of Pseudomonas pseudomallei. Antimicrob Agents Chemother 4, 193–5 (1973). 4790939 10.1128/aac.4.2.193 PMC444526

[b29] Sarkar-Tyson M., Smither S. J., Harding S. V., Atkins T. P. & Titball R. W. Protective efficacy of heat-inactivated B. thailandensis, B. mallei or B. pseudomallei against experimental melioidosis and glanders. Vaccine 27, 4447–51 (2009). 19490962 10.1016/j.vaccine.2009.05.040

[b30] Ulrich R. L., Deshazer D., Brueggemann E. E., Hines H. B., Oyston P. C. & Jeddeloh J. A. Role of quorum sensing in the pathogenicity of Burkholderia pseudomallei. J Med Microbiol 53, 1053–64 (2004). 15496380 10.1099/jmm.0.45661-0

[b31] Nelson M., Prior J. L., Lever M. S., Jones H. E., Atkins T. P. & Titball R. W. Evaluation of lipopolysaccharide and capsular polysaccharide as subunit vaccines against experimental melioidosis. J Med Microbiol 53, 1177–82 (2004). 15585494 10.1099/jmm.0.45766-0

[b32] Hassan M. R., Pani S. P., Peng N. P., Voralu K., Vijayalakshmi N., Mehanderkar R., Aziz N. A. & Michael E. Incidence, risk factors and clinical epidemiology of melioidosis: a complex socio-ecological emerging infectious disease in the Alor Setar region of Kedah, Malaysia. BMC Infect Dis 10, 302 (2010). 20964837 10.1186/1471-2334-10-302 PMC2975659

[b33] Currie B. J., Jacups S. P., Cheng A. C., Fisher D. A., Anstey N. M., Huffam S. E. & Krause V. L. Melioidosis epidemiology and risk factors from a prospective whole-population study in northern Australia. Trop Med Int Health 9, 1167–74 (2004). 15548312 10.1111/j.1365-3156.2004.01328.x

[b34] Sivalingam S. P. Sim S. H. Jasper L. C. Wang D. Liu Y. & Ooi E. E. Pre- and post-exposure prophylaxis of experimental Burkholderia pseudomallei infection with doxycycline, amoxicillin/clavulanic acid and co-trimoxazole. J Antimicrob Chemother 61, 674–8 2008. 18192684 10.1093/jac/dkm527

[b35] Craig W. A. Pharmacodynamics of antimicrobials: general concepts and applications. in: C. H. Nightingale, T. Murakawa, and P. G. Ambrose, (Eds.), Antimicrobial pharmacodynamics in theory and clinical practice., Marcel Dekker, Inc. New York, NY, 2002, pp. 1–22.

[b36] Newton P. N., Chaulet J. F., Brockman A., Chierakul W., Dondorp A., Ruangveerayuth R., Looareesuwan S., Mounier C. & White N. J. Pharmacokinetics of oral doxycycline during combination treatment of severe falciparum malaria. Antimicrob Agents Chemother 49, 1622–5 (2005). 15793155 10.1128/AAC.49.4.1622-1625.2005 PMC1068593

[b37] Heine H. S., Louie A., Sorgel F., Bassett J., Miller L., Sullivan L. J., Kinzig-Schippers M. & Drusano G. L. Comparison of 2 antibiotics that inhibit protein synthesis for the treatment of infection with Yersinia pestis delivered by aerosol in a mouse model of pneumonic plague. J Infect Dis 196, 782–7 (2007). 17674322 10.1086/520547

